# Predicting acceptance and adoption of renewable energy community solutions: the prosumer psychology

**DOI:** 10.12688/openreseurope.14950.1

**Published:** 2022-09-29

**Authors:** Francois Brambati, Daniele Ruscio, Federica Biassoni, Rebecca Hueting, Alessandra Tedeschi

**Affiliations:** 1 Deep Blue, Rome, Italy; 2 Università Cattolica, Milan, Italy

**Keywords:** Prosumer, Social Acceptance, Renewable Energy Technology, Behavioural Intention, Environmental risk perception

## Abstract

**Background:** This paper, in the frame of social acceptance of renewable energies and innovative community-based production and consumption models, aims at supporting a data-driven approach able to deal with climate change and identify and quantify the psycho-sociological dimensions and factors that could support the transition from a technology-driven approach to a consumer-driven approach throughout the emerging “ *prosumer* business models”. In addition to the already existing Social Acceptance dimensions, this paper tries to identify a purely individual psychological fourth dimension to understand the processes and factors that underlie individual acceptance and adoption of new renewable energy business models, with the realization of a Prosumer Acceptance Index.

**Methods:** Questionnaire data collection has been performed throughout an online survey platform, combining standardized and ad-hoc questions adapted for the research purposes, based on the developed theoretical model.

To identify the main factors (individual/social) influencing the relation with renewable energy technology adoption, a Factorial Analysis has been conducted to identify the latent variables that are related to each other. Linear regression has been conducted to identify and quantify the factors that could better predict behavioural intention to become a prosumer.

**Results:** Five latent psychological factors were revealed: concern about environmental issues, interest in energy sharing, concern on climate change, social influence and impact on bill cost. Three variables were found to significantly measure and predict the scores of the “Acceptance in becoming a prosumer” ad hoc scale: attitude, economic incentive and age.

**Conclusions:** This research can facilitate policymakers and stakeholders to better understand which relevant psycho-sociological factors are intervening in the renewable energy technology acceptance processes and what and how specifically target when proposing change towards sustainable energy production and consumption.

## Introduction

### Change renewable energy business model?

Climate change is considered by Europeans as the single most severe problem facing the world ( Eurobarometer, 2021). To mitigate climate change, it is crucial to transit from fossil to renewable energy sources ( EU Climate Pact, 2020; Liu *et al.*, 2020). The success of such transition strongly depends also on efficiency and effectiveness in the adoption of renewable energy business models in local and national communities ( de Coninck *et al.*, 2018; Devine-Wright, 2007; Devine-Wright, 2009; Liu *et al.*, 2020; Papazu, 2017; Wüstenhagen *et al.*, 2007). Renewable energy communities are going to be a reality in Europe soon, pushed by the recent Clean Energy Directive approved in late 2019. This directive obliges Member States to ensure a more competitive, customer-centred, flexible, and non-discriminatory EU electricity market with market-based supply prices. It strengthens existing customer rights, introduces new ones, and provides a framework for energy communities of *prosumers*. Currently, Member States are working on the transposition of the Directive into national regulations. Public acceptance of renewable energy and “renewable energy communities” is of increasing concern to policymakers in many countries, who aim to mitigate climate change by rapidly and extensively increasing the deployment of renewable energy technologies ( Devine-Wright, 2011). Several policymakers are increasing their commitment towards sustainability and several Research and Innovation projects are purposely focusing on direct consumers’ engagement in the energy transition. Different business models for sustainable community energy production and consumption can be identified, and amongst them, RENAISSANCE identified a set that we can call “Prosumer business models” ( RENAISSANCE, 2020).

### Prosumer business models

The *Prosumer* term, a combination of “producer” and “consumer”, was first credited to Alvin Toffler in *The Third Wave* ( Stewart, 2014; Toffler, 1980). After the Modernist culture of production, and Postmodernist culture of consumption, today the aware, active, and technologically engaged *prosumer* is on the rise ( Comor, 2011) and it is challenging the consumer society’s division of consumers and producers ( Tobiassen, 2008). This research, to define the *prosumer*, apply the working definition from von Hippel (1986): “what distinguishes prosumers is their advanced technological skills and that they use these skills to produce as well as consume”. Specifically, the term *prosumer* here will be used in relation to new renewable energy business models. But how is it possible to facilitate the generation and acceptance of *prosumer models* in a society, in order to facilitate business models for sustainable energy production and consumption? The acceptance and adoption of renewable energy technology (RET) models from individuals, members of specific communities intended in becoming *prosumers*, relies on two main pillars: social acceptance and individual acceptance.

### Achieve a prosumer community: path to social acceptance

**Figure 1.**
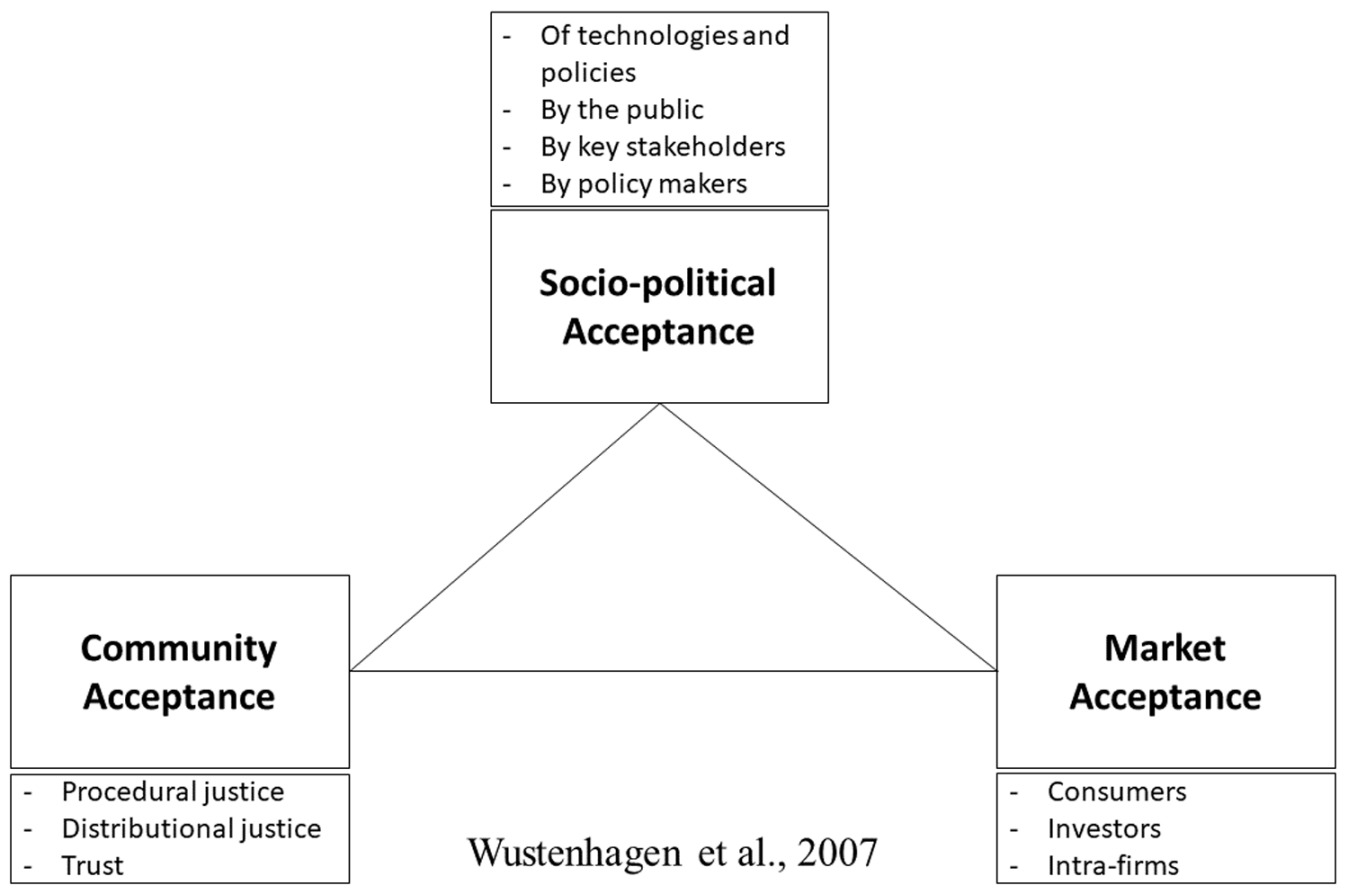
Social acceptance ( Wüstenhagen *et al.*, 2007).

Many researchers have argued that social and culturally relevant issues are critical barriers to RETs ( Claudy *et al.*, 2010; Sovacool, 2009a; Sovacool, 2009b; Zoellner *et al.*, 2008). Carlman (1984) was the first scholar that defined the problem of social acceptance (for wind power) and stated that siting wind turbines were ‘‘also a matter of public, political, and regulatory acceptance’’ ( Carlman, 1984). The concept of Social Acceptance of renewable energy innovation was formalized by Wüstenhagen *et al.* (2007), presenting three dimensions of the concept ( Figure 1).

**Socio-political acceptance**: on the broadest, most general level ( Wüstenhagen *et al.*, 2007). Both policies (e.g., ecological tax reform, European Clean Energy Directive, 2019) and technologies represent successful cases of societal acceptance.

**Community Acceptance**: refers to the specific acceptance of siting decisions and renewable energy projects by local stakeholders, particularly residents and local authorities ( Wüstenhagen *et al.*, 2007). A particular feature is that it has a time dimension. As Wolsink (2007) demonstrates, the typical pattern of local acceptance follows a U-curve, going from high acceptance to (relatively) low acceptance during the siting phase (usually still positive on average) and back up to a higher level of acceptance once a project is up and running. 

**Market Acceptance:** the process of market adoption of an innovation ( Wüstenhagen *et al.*, 2007). Consumers have changed, becoming investors in distributed energy. The ownership of renewable energy devices, such as solar panels, becomes a question ( Zhai & Williams, 2012). In this perspective, we can learn from the literature on diffusion of innovation ( Rogers, 1995), which explains the adoption of innovative products by consumers through a communication process between individual adopters and their environment.

## The specificity of RETs

The number of RETs features imply the existence of diversified sets of barriers to their Social Acceptance. For instance, renewable energy plants tend to be smaller scale than conventional power plants, increasing the number of siting decisions that need to be taken ( Wüstenhagen *et al.*, 2007) becoming an individual investment decision. Furthermore, renewable energy conversion tends to be characterized by lower energy densities while the relative visual impact tends to be higher ( Wüstenhagen *et al.*, 2007). This is in part reinforced by the fact that resource extraction of fossil or nuclear energy happens below the earth’s surface ( Sieferle, 1982) and is invisible for the everyday life of a citizen. At last, most RETs do not compete with actual technologies making their acceptance a choice between short-term costs and long-term benefits ( Wüstenhagen *et al.*, 2007). The need to take more micro-level siting decisions impacts - among other issues - the need to preserve the aesthetic dimension, the sense of ownership of the local area, the need for security, and economic investment uncertainties. To avoid getting to the situation where people hinder the adoption of RETs, known as Not in my backyard (NIMBY), where residents oppose to proposed developments in their local area supporting strict land-use regulations, a consumer-driven approach to socially accept RETs implies community and group understanding to result in its activation. As Wüstenhagen *et al.* (2007) noted, once more, RET acceptance is a collective action problem, also known as a social dilemma, which is a choice between short-term costs and long-term benefits.

**Figure 2.**
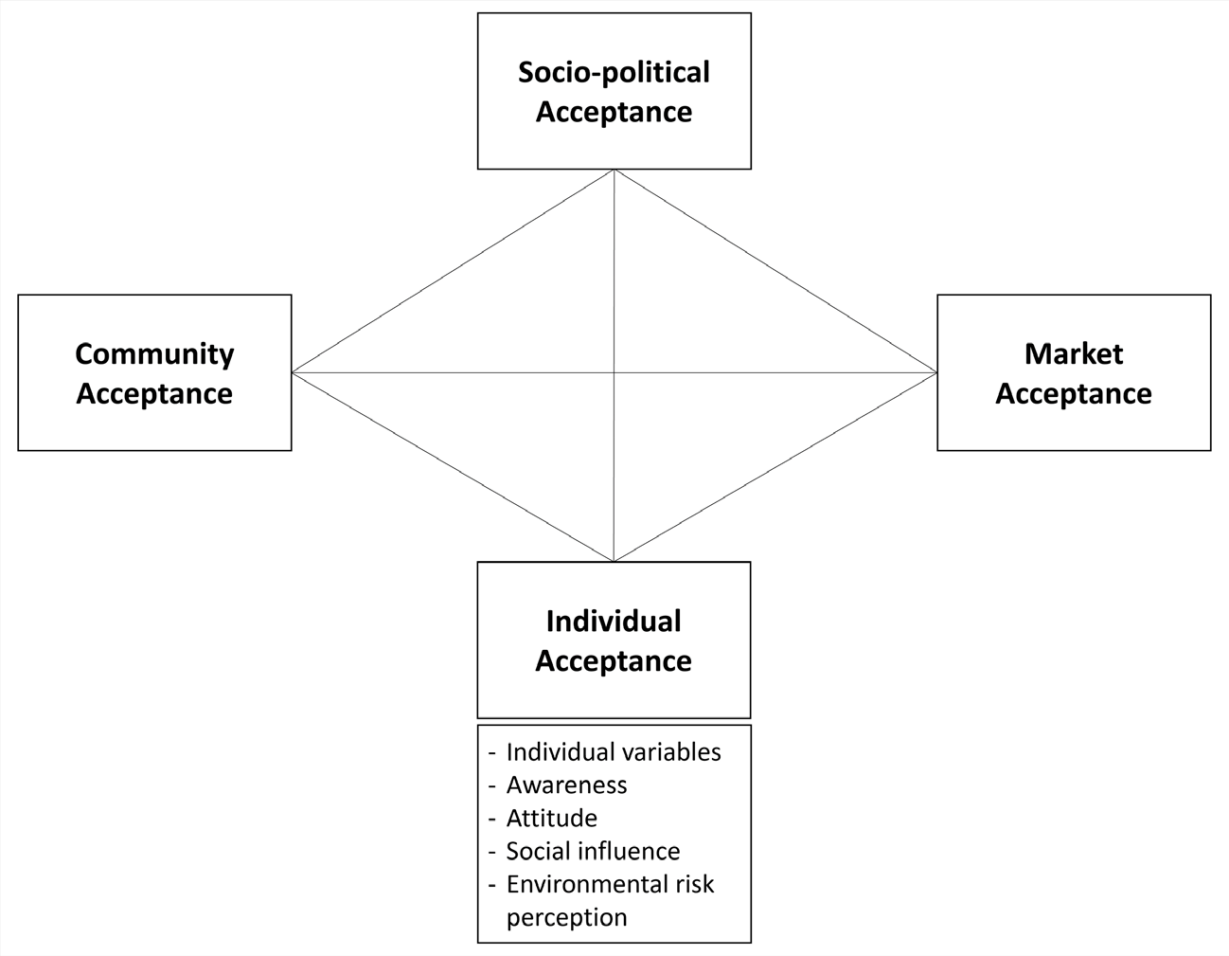
Social acceptance 4th dimension: individual acceptance.

For these reasons, to achieve the understanding of a *prosumer* community it could be relevant to add a fourth dimension ( Figure 2) which includes all individual cognitive, affective, and motivational factors, that can help explain people’s decision-making process in adopting renewable energy technologies. In psycho-social literature, a certain number of communities’ and groups’ factors have been found to have an influence on the individual community member.

Social norms are formed in group situations and subsequently serve as standards for the individual’s perception and judgment ( Sherif & Sherif, 1953). Norms not only detail what is appropriate behavior, but the expectations define what the group does, and who the group is. Deviation from social norms leads to engender conformity ( Festinger, 1950). This tendency to conform is a consequence of Social Influence, or as the process wherein one person’s attitudes, cognitions, or behaviors are changed through the doings of another ( Cialdini & Griskevicius, 2010). A Nation interested in spreading sustainable and positive behaviors would want to share with its citizens’ values and norms on global and environmental issues. That feeling of being part of the same group, and sharing those behaviors and values, generally, bring individuals to help each other and to cooperate for common objectives. Cooperation is a behavior part of a range of behaviors identified as Prosocial Behaviours, which are often associated with morality ( Baron, 1997; Batson *et al.*, 2002; Staub, 1978); this includes several behaviors such as helping, sharing and cooperating ( Batson, 1998). According to Cialdini (1991), individuals may act prosocially to benefit others or themselves. Finally, a series of other factors, like national identity, can influence RETs’ acceptance. National identity, as a person’s identity or sense of belonging to one state or one nation ( Ashmore *et al.*, 2001), is not an inborn trait and is essentially socially constructed ( Anderson, 1991). People incorporate national identity into their personal identities by adopting beliefs, values, assumptions, and expectations, if personally meaningful, translating them into daily practices ( Ashmore *et al.*, 2001; Bar-Tal & Staub, 1997; Hogg & Reid, 2006).

### Individual acceptance: what does psychology say about prosumers?

To understand the way individual factors can explain who the *prosumer* is and how they behave, fundamental is to introduce a set of behaviors known in the field of sustainable energies and environmental issues as pro-environmental behaviors (PEBs). When defined by its intent, PEB is a behavior undertaken with the intention to change the environment from the actor's standpoint ( Huang, 2016; Stern, 2000). However, how the behavioral intention to adopt a prosumer model can be described? While the majority of benefits are for the environment and society as a whole, PEBs frequently imply personal costs. Value has been identified as a significant antecedent of PEBs ( Abrahamse & Steg, 2013; Nordlund & Garvill, 2002; Steg *et al.*, 2009; Steg *et al.*, 2015). It has been demonstrated that biospheric values (caring about the preservation of the natural world) are particularly crucial ( Steg & De Groot, 2012; Steg *et al.*, 2014b). Biospheric values are highly and positively correlated with PEBs when compared to other values ( Nordlund & Garvill, 2002; Steg *et al.*, 2011; Steg *et al.*, 2014a). Being aware of global climate change may motivate people to act ecologically friendly behaviours, even when relatively expensive ( Loebnitz & Aschemann-Witzel, 2016; Steg, 2016; Steg *et al.*, 2014a; Verplanken & Holland, 2002). However, it is unclear if these values could also serve as the driver motivation to become a *prosumer*. Environmental self-identity ( van der Werff *et al.*, 2013b) is another variable that could help to explain the connection between biospheric values and PEBs. It measures how much individuals believe themselves as someone who acts in a sustainable way ( van der Werff *et al.*, 2013a), and in order to be or look consistent, people are compelled to act in accordance with how they perceive themselves ( Bem, 1972). In fact, stronger the self-identity to protect the environment, stronger is the tendence to recycle ( Mannetti *et al.*, 2004; Whitmarsh & O'Neill, 2010), participate in environmental activism ( Fielding *et al.*, 2008), use fuel-efficient vehicles and sustainable modes of transportation ( van der Werff *et al.*, 2013b), plan to eat less meat ( van der Werff *et al.*, 2013b), and prefer environmentally friendly products ( Barbarossa & De Pelsmacker, 2016; van der Werff *et al.*, 2014)

***How to support social and individual acceptance to support Prosumer?*** In order to combine social and individual acceptance on the behavioral intention of becoming a *prosumer*, different psychological theories can be applied. A consolidated starting point can be considered the Theory of Planned Behaviour (TPB; Ajzen, 1991) which assesses someone’s behavioral intention to engage a specific behavior (adopting a RET) is influenced by subject’s **attitudes**; **personal norms**; and **perceived behavioral control** ( Ajzen, 1991). While TPB could explain the intention of becoming a *prosumer*, it couldn’t fully explain people’s attitudes and perceived behavioral control in accepting new technological business models such as RETs. Davis's technology acceptance model (TAM; Davis, 1989; Davis *et al.*, 1989) included the technological aspects in the TPB framework. TAM shares strong behavioral elements, although, intentions are meant as perceived usefulness and Perceived ease-of-use. Meaning that people need to perceive usefulness and intuitive usability in the RET other than having favorable attitudes to adopt it. Expanding further TAM, attitudes can then be also declined into **Environmental Attitude** (EA). EA has been defined by Hines *et al.* (1987) as “the individual’s feelings, favorable or unfavorable, about particular aspects of the environment or objects related to the environment”. Finally, Explanatory behavior theories (e.g. Stern, 2000) linked environmental values and people's attitudes, which again influence their intention and behavior, while the Norm Activation Model (NAM; Schwartz & Howard, 1981) provided empirical support explaining prosocial behaviors within local and community environmental contexts and energy conservation ( Black *et al.*, 1985; Tyler *et al.*, 1982); willingness to pay for environmental protection ( Guagnano, 2001; Guagnano *et al.*, 1994); recycling ( Bratt, 1999; Hopper & Nielsen, 1991; Vining & Ebreo, 1992); and general pro-environmental behavior ( Nordlund & Garvill, 2002; Schultz *et al.*, 2005). This research highlighted how, in the formation of a personal norm, two other factors should be included in “behavioral intention” models, namely: **Awareness of consequences** and **control believes**. Rotter (1966) Locus of control theory (LOC) refers to an individual's perception about the underlying main causes of events in their life. If people think events can be driven by their behaviors that is a belief in internal control. Attributing internal causality, although, is not enough to engage a behavior. Bandura in his Social Cognitive Theory (1977) affirms that the inclination to engage in a given behavior is strongly influenced by self-efficacy, as the confidence one has in the ability to perform the behavior, and by the expected outcomes, as the expectation, often rewarding, of acting a behavior. Finally, another significant dimension worth mentioning is the concept of Environmental risk perception. RETs are characterized by a high level of uncertainty, with long-term and delayed consequences, and also consequences that occur having RETs in the community’s backyard. All these aspects may result in a discounting of such risks, which are taken less seriously than risks with negative outcomes that occur for sure, now and here ( Gattig & Hendrickx, 2007). When dealing with environmental risks, it is frequently necessary to balance benefits that occur for sure and immediately against losses that are uncertain and delayed ( Gattig & Hendrickx, 2007). Decision-making, influenced by opting between pro-environmental and pro-self-alternatives, concerns the trade-off between risks and benefits of adopting renewable energies and is an essential step towards social acceptance. This trade-off may be modulated by motivational factors, such as intrinsic and extrinsic sources of motivation ( Ryan & Deci, 2020). While both sources can motivate PEBs, extrinsic motivation can also have an inhibiting effect on the self-driven motivations of “green” consumers ( Ali *et al.*, 2020). Individual differences in environmental concern are viewed as an intrinsic motivational factor that influences willingness to accept larger trade-offs between personal and environmental gain ( Hansla *et al.*, 2008). External motivational factors, such as heuristics ( Tversky & Kahneman, 1974) can be represented by a tendency to make judgments close to anchors, as described by Tversky and Kahneman (1974). For example, anchoring influences people’s willingness to accept and pay for carbon taxes for flying, even when people have been made aware of the influence from anchors on decisions beforehand ( Bahník *et al.*, 2016; Brouwer *et al.*, 2008; Furnham & Boo, 2011; Sonnenschein & Smedby, 2019; Strack *et al.*, 2016). Silvia (2008) highlights the benefits of motivating the *prosumer*, but the literature stops short of identifying what are the variables that would motivate the *prosumer*.

**Figure 3.**
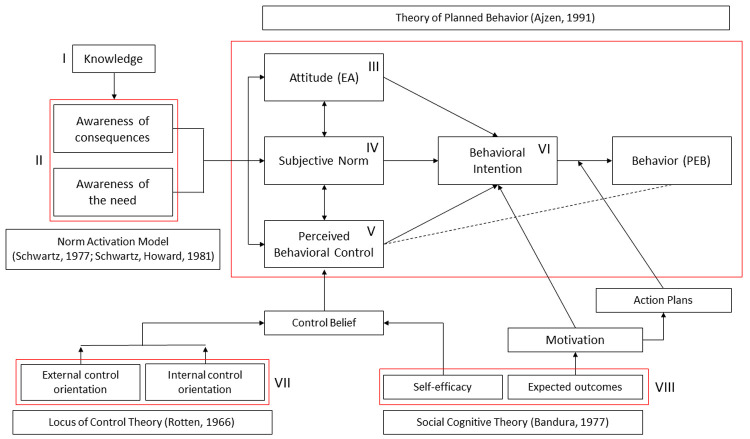
Integrated Prosumer Theoretical Model. The roman numbers indicate the specific models and variables that were operationalized in the final form of the survey.

***The present study*.** The present research aims to support a data-driven approach able to deal with climate change and identify & quantify the psycho-sociological dimensions and factors that could facilitate the transition throughout the “ *prosumer* business models” ( Figure 3). Starting from all the theoretical definitions of the *prosumer* psychological individual and social characteristics leading to social acceptance of this new renewable energy business model, the main models emerged from the literature review will be investigated and quantified, in a sample of European citizens, to extract the main factors able to influence the decision of becoming a *prosumer* (i.e., intentional acceptance to adopt a RET). The specific goals were twofold: 1) Identify the main factors (individual and social) behind the adoption of being a *prosumer* and quantify their importance (for the ones that were already *prosumers*); 2) Identify and quantify the factors that could be better able to predict the decision to become a *prosumer* (for the ones that are not yet *prosumers*). In that way, the present research aims to facilitate policymakers and relevant stakeholders to better understand the relevant psycho-sociological factors to specifically target when proposing change towards sustainable energy production and consumption.

## Methods

To understand which socio-psychological factors could define social acceptance of RET and innovative community-based production and consumption models (the *Prosumer* business models for local communities), an online survey was set up and administered to consider, according to the literature review, the most relevant aspects influencing Individual and Social Acceptance from local communities ( Figure 3).

### Building the “Prosumer” Acceptance Index survey

The first reference framework for building the questionnaire was Wüstenhagen *et al.* (2007), and the 3 main sub-components identified in forming the so-called “triangle of social acceptance” with the addition of the 4th component regarding individual psychological factors. Items coming from already validated scales, like the Awareness towards social acceptance factor ( Wüstenhagen *et al.*, 2007) were used to assess respondents' perception and knowledge about energy loss in energy transportation, about which energies could be defined as renewable, which energy system is most consuming and knowledge about European directives. From Ellis & Ferraro (2016) and Huijts *et al.* (2012), the most relevant aspects influencing social acceptance were derived. Questionnaire developed by Moula *et al.* (2013), was also included, complemented with additional ad-hoc questions to cover all the dimensions. To have an idea of the personal differences in how people accept and use technology, Davis’s (1989) technology acceptance model was implemented as an integration of Ajzen’s (1991) Planned Behaviour Theory. Ad hoc questions were also included to determine the perceived risks and benefits of specific *prosumer* business models proposed. An initial version of the survey was generated combining standardized and ad-hoc questions, adapted for the purposes of the present research. The final survey was composed of 31 main questions: 5 multiple-choice questions, 12 items requiring the respondent to declare the level of agreement on a 5-point Likert scale, 6 items that required the respondent to rank the order of perceived importance of different elements, 7 close-ended and 2 open-ended responses ( Table 1). For the close-ended responses a numerical score was calculated, assessing 1 point to the correct answers. The final survey was divided into three parts: The first part introduced the project, the aims of the survey and ethics statements regarding the privacy policy, consent form, and GDPR compliance pages and information. No personal or sensitive information was collected; the data was anonymized from the beginning. The second part collected 10 socio-demographic variables (such as gender, age, nationality, level of education etc.). This information was analyzed in an aggregated and anonymized way. The third part collected questions about the following factors: knowledge (I) and awareness of renewable energies (II); individual environmental attitudes (III); personal norms influences (IV); individual decision making and perceived behavioral control (V) as the perceived control orientation (VI) and expected outcomes (VII) to accept RETs, and relative perceived risks and benefits; and a series of questions to quantify the intention to accept the *prosumer* sustainable business model proposed by EU regulation.

**Table 1.** Structure of the “ *prosumer*” Index Survey.

Independent Variables ( Figure 3)		*Example of Item*
Demographic	1. Age (Q1) 2. Gender (Q2) 3. Country last five years (Q3) 4. Type of environmental context (i.e., Rural/Urban) (Q6) 5. Level of education (Q7) 6. Current typology of consumer (Q8) 7. Annual net income (Q9)	*Among the following energy * *consumer types, which one best * *represents your current position?*
Awareness (I; II;)	8. Awareness on energy loss (Q12-1; Q12-2; Q12-3; Q12-4; Q12-5; Q12-6; Q12-7) 9. Awareness on renewable energies (Q13-1; Q13-2; Q13-3; Q13-4; Q13-5; Q13-6; Q13-7; Q13-8; Q13-9; Q13-10) 10. Awareness energy consumption (Q14-1; Q14-2; Q14-3; Q14-4; Q14-5; Q14-6) 11. Awareness on European directives (Q24)	*Among the following energy sources, * *please select the ones you think are * *renewable.*
Attitudes (II; III)	12. Concern Climate Change (i.e., biospheric values) (Q15-1; Q15-2; Q15-3; Q15-4; Q15-5; Q15-6; Q15-7; Q15-8; Q15-9) 13. Concern Environmental Issues (Q16-1; Q16-2; Q16-3; Q16-4; Q16-5; Q16-6; Q16-7; Q16-8; Q16-9; Q16-10) 14. Impact of current energy production on environment (Q17) 15. Attitude of using a RET (Q18) 16. Attitudes on local production of renewable energies (Q19) 17. Attitudes on perceived level of control in Local / Community decisions (Q22; Q74-v; Q75-v; Q76-v) 18. Perceived importance of directives to build strategy toward ren energies (Q25) 19. Most to least favourite production system (Q46; Q146-v; Q149-v)	*Among the following environmental * *issues, which ones are of most * *concern in your opinion, on a global * *scale?*
Social Norms (IV, VII)	20. Ask advice to switch supply [Internal Social Norm - Individual] (Q41) 21. Adopt ren energies if the neighborhood do [External Social Norm - Conformity] (Q42)	*How likely would it be for you to * *ask for advice before switching to a * *different energy supply service?*
Economic Control and Environmental Risk Perception (V, VIII)	22. Perceived Economic cost of renewable energies (Q33; Q90-v) 23. Individual and societal Economic costs/benefits of switching to renewable-only energy providers (Q34-1; Q34-2; Q34-3) 24. Risks to acceptance renewable own supply (Q35; Q95-v; Q96-v; Q97-v) 25. Risks to acceptance renewable own property (Q36; Q99-v; Q100-v; Q101-v; Q102-v; Q103-v) 26. Risks to acceptance renewable own community (Q37; Q105-v; Q106-v; Q107-v; Q108-v; Q109-v; Q110-v; Q111-v) 27. Benefits to acceptance renewable own supply (Q38; Q113-v; Q114-v; Q115-v; Q116-v; Q117-v) 28. Benefits to acceptance renewable own property (Q39; Q119-v; Q120-v; Q121-v; Q122-v; Q123-v) 29. Benefits to acceptance renewable own community (Q40; Q125-v; Q126-v; Q127-v; Q128-v; Q129-v)	*Using existing renewable energy * *technologies may result in a less * *expensive bill for the consumers who * *adopt them.*
**Dependent Variable**		
Acceptance of Being a Prosumer (VI)	30. (Individual) Acceptance of renewable energy system own property (Q44- 1; Q44-2; Q44-3) 31. (Social) Acceptance of renewable energy system own community (Q45-1; Q45-2)	*To what extent would you agree * *to install a small to medium size * *renewable energy production system * *on your property*

### Procedure

All the questions in the second and third part of the survey were mandatory, so the respondent could not proceed with the survey if some item was not responded to, but to partially mitigate this effect, the option “other” and open-ended questions were always inserted, to enable the respondent to express additional information or comments and integrate their responses. The data collection was completed between 28th April 2020 and 31st July 2020 with the translation and distribution in seven different European languages (Italian, English, French, Dutch, Spanish, Greek, Polish). The online survey was distributed via the Survey Monkey platform.

### Sample

A total of 212 respondents from a total of 17 nations answered the survey, aged from 18 to over 65 (female = 57%; N = 121; male = 42%; N = 89; other = 1%; N = 2). Age distribution presented more than 10 responders for each age range, and it reached almost the 50% percentile by responders younger than 34 years (cumulate percentage: 46%) while the rest of responders can be described as older than 34 years old (cumulate percentage: 54%). Almost all respondents’ education levels varied between bachelor’s degrees and Ph.D. (Cumulative Percentage: 90%). More precisely, “College diploma/bachelor’s degree or higher” is, by far, the most frequent category (Mode: N=170), followed by “Doctorate” (N=20) and “Secondary school/Upper secondary” (N=18). The full sample is composed almost entirely of household consumers (Mode: N=128), tenants/leasehold consumers (N=57), and landowner consumers (N=21): all categories with similar power behaviors. No industrial or commercial consumers participated in the survey. Respondents of the survey formed a heterogeneous sample, including countries like Germany, The Netherlands, the United Kingdom, Belgium, Switzerland, Poland, France, Albania, Slovenia, Hungary, Italy, Bulgaria, Portugal, Spain, Greece.

### Ethics and consent

All RENAISSANCE results have been conducted according to the requirements of ethics and integrity of the GA.

An Ethics Issues Table has been completed as part of the application process in addition to the Ethics Self-Assessment form. Guidelines and instructions by the EC have been followed and all ethics obligations have been fulfilled.

Deliverables D8.1 (POPD – Requirements n. 1), D8.2 (POPD – Requirement No. 2) and D. 1.4 (Data Management Plan) are used as obligatory guidelines for the project implementation, the data collection and the deliverables development.

Participants were asked to give consent before starting the survey by submitting a form; the consent was voluntary and renegotiable, so participants could decide to withdraw at any point.

### Data analysis

Internal consistency of the “prosumer index survey” was tested using Cronbach’s alpha for each of the psychological factors highlighted in Table 1, while for the socio-demographic data of the sample, descriptive statistics were performed.

To identify the main factors (individual and social) modeling the relation with renewable energy technology models for participants to the survey that were already *prosumers*, a Factorial Analysis has been conducted to identify the latent variables that are related to each other, to generate a data-driven model of a “ *prosumer*”.

To identify and quantify the factors that could better predict the behavioral intention to become (or not become) a *prosumer*, linear regression has been conducted to estimate the influence of the independent variables on the dependent variable Acceptance of being a *Prosumer* ( Table 1).

Data processing and analysis were performed through the IBM SPSS Statistics 21.

The carried out analyzes between the different variables took into account an acceptability confidence interval of 95%.

## Results

### Cronbach’s alpha and descriptive statistics

For the groups of items addressing “Awareness” the coefficient for the items was .778. For the groups of items addressing “Attitudes”, the alpha coefficient was .636. For the groups of items addressing “Social Norms”, the alpha coefficient was .631. For the groups of items addressing the “Economic Control” part of the “Environmental Risks”, the alpha coefficient was respectively .577, while for the remaining items, they were ranking questions, so no overall score could have been considered. Given the acceptable alpha coefficient, for each factor, a sum of the items was calculated, for each independent variable ( Table 2).

**Table 2.** Descriptive Statistics.

		Awareness	Attitudes	Social Norms	Environmental Risk	Being a Prosumer
N	Valid	197	182	131	154	130
	Missing	15	30	81	58	82
Mean		5,38	61.54	7.89	18.02	8.81
Std. Deviation		1,39	9.19	1.97	4.75	1.42
Skewness		-.74	-1.05	-1.18	-1.01	-1.86
Std. Error of Skewness		.17	.18	.21	.20	.21
Kurtosis		.48	1.62	1.15	.56	5.36
Std. Error of Kurtosis		.35	.36	.42	.39	.42
Minimum		1	23	2	4	2
Maximum		8	77	10	25	10
Percentiles	25%	4	56	7	16	8
	50%	6	62.5	8	19	9
	75%	6	68	9	21	10

### Factorial analysis

A factorial using principal axis factoring as the extraction method, with a Varimax with Kaiser normalization rotation method was used, entering all the items concerning psycho-sociological dimensions considered on the survey, for all the users that were considered as “Potential Prosumers” (N=112). The threshold for being a *prosumer* was set using percentile variability in the summed scores of the “Acceptance of being a prosumer” higher than average 3 on a 5-point average Likert scale, as well as the scores to those features that already characterize a *prosumer* (i.e., Q8).

Kaiser-Meyer-Olkin measure for collinearity was good (KMO=.825) as the Bartlett’s Test of Sphericity (sig. .000). As reported in Table 3, the extracted communalities were all above .400, except for Q34-3 (.384) that was close to.400 and so was kept, while Q16-9 (.347) and Q33 (.180) were removed from the analysis.

**Table 3.** Communalities.

	Initial	Extraction
Q15-1 Concern Climate Change	.640	.640
Q16-1 Acidification of rain and oceans	.611	.605
Q16-2 Air pollution	.574	.585
Q16-3 Rising of temperatures	.660	.818
Q16-4 Extreme weather conditions	.449	.423
Q16-5 Environmental resource exploitation	.556	.531
Q16-6 Loss of biodiversity	.521	.504
Q16-7 Pollution of rivers and seas	.641	.673
Q16-8 Soil pollution	.643	.686
Q16-10 Waste disposal	.466	.470
Q41 Internal Social Norm - Individual	.334	.514
Q42 External Social Norm - Conformity	.451	.573
Q34-1 Less expensive bills	.465	.643
Q34-2 Unvaried bills	.581	.808
Q34-3 Higher bills	.342	.384
Q44-1 For own consumption	.435	.428
Q44-2 For an organised form of shared consumption	.498	.632
Q44-3 To sell the extra amount to the general electricity grid for the market	.551	.601
Q45-1 To sell the extra amount to the general electricity grid and get a discount on your monthly bill.	.562	.596
Q45-2 for the collective consumption of the local community.	.564	.622

Extraction Method: Principal Axis Factoring.

**Figure 4.**
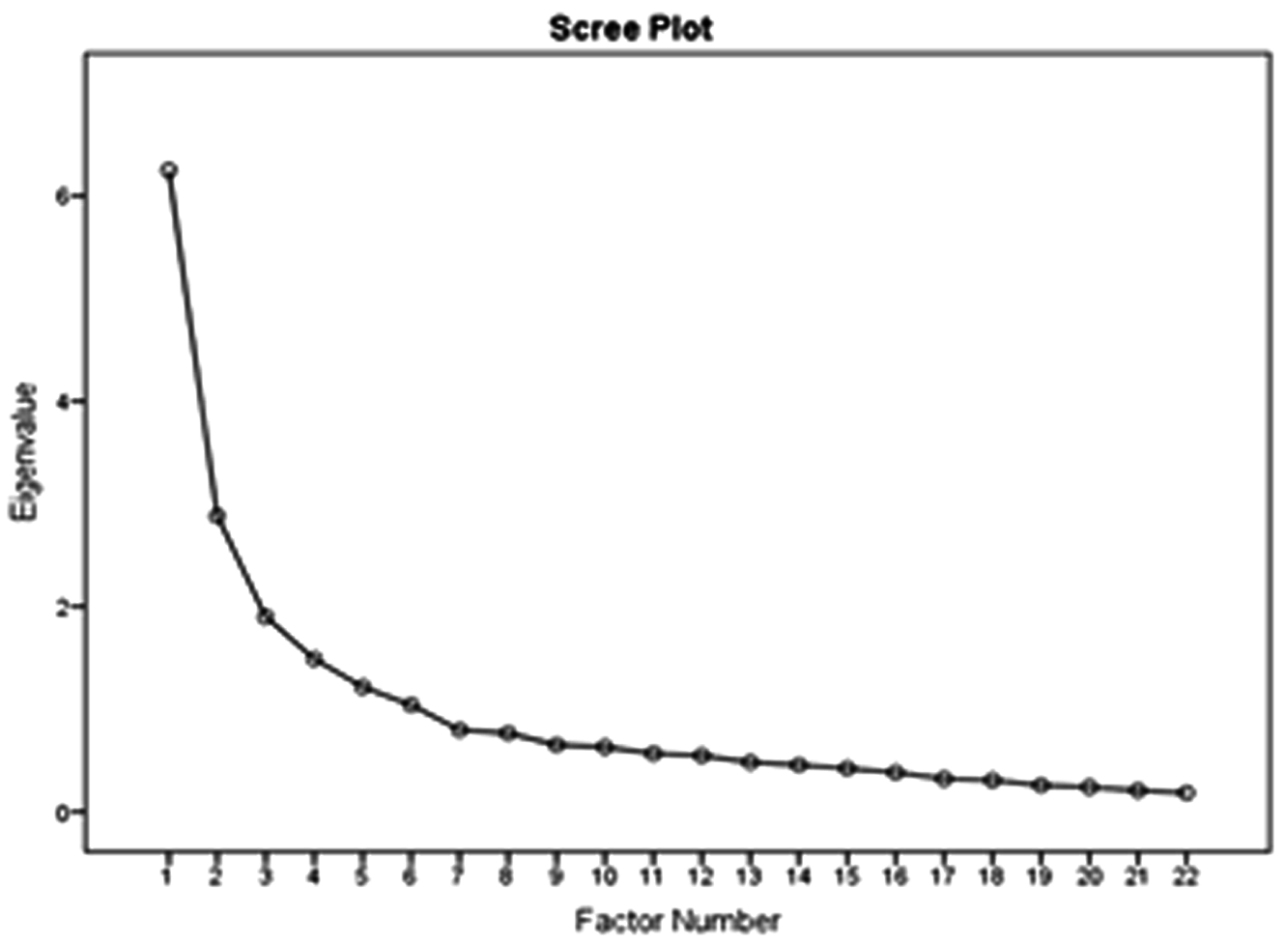
Plot of Eigenvalues as a function of the number of principal components retained in the model.

As it can be seen in Figure 4 and Table 4, the analysis yielded five factors explaining a total of 62.428% of the total variance for the entire set of variables. The *prosumer* could be then described by those 5 latent variables.

**Table 4.** Factors.

Items	Rotated Factors Matrix ^( a)^				
	1. Concern about Environmental Issues	2. Interest in Energy Sharing	3. Concern on Climate Change	4. Social Influence	5. Impact of Costs on Bills
Q16-8 Soil pollution	0.820				
Q16-7 Pollution of rivers and seas	0.815				
Q16-1 Acidification of rain and oceans	0.700				
Q16-2 Air pollution	0.682				
Q16-10 waste disposal	0.647				
Q16-5 Environmental resource exploitation	0.647				
Q16-6 Loss of biodiversity	0.617				
Q45-2 for the collective consumption of the local community.		0.713			
Q44-2 for an organised form of shared consumption with the local community.		0.701			
Q44-3 to sell the extra amount to the general market electricity grid.		0.644			
Q44-1 for own consumption.		0.650			
Q45-1 to sell the extra amount to the general electricity grid and get a discount on your monthly bill.		0.676			
Q16-3 Rising of temperatures	0.460		0.749		
Q15-1 Concern Climate Change	0.508		0.59		
Q16-4 Extreme weather conditions	0.455		0.427		
Q41-Ask advice to switch supply [Internal Social Norm - Individual]				0.690	
Q42 Adopt ren energies if the neighborhood do [External Social Norm - Conformity]				0.650	
Q34-1 Less expensive bills					0.777
Q34-2 Unvaried bills					0.640
Eigenvalues:	6.249	2.881	1.898	1.490	1.216
% of Total Variance	28.40	13.09	8.62	6.77	5.52
**Total Variance**					**62.43%**

Extraction Method: Principal Axis Factoring. Rotation Method: Varimax with Kaiser Normalization. (a) Rotation converged in 8 iterations.

Factor 1 was labeled “ *Concern about Environmental Issues*” due to the high loadings of the following items: concerns on climate change (Q15.1); concerns on acidification of rain and oceans (Q16.1); on air pollutions (Q16.2); on waste disposal (Q16.10); environmental resource exploitation (Q16.5); loss of biodiversity (Q16.6) and concerns on the effects of traffic congestion on environment (Q16.9). This first factor alone explained 28.403% of the total variance.

Factor 2 was labeled “ *Interest in Energy Sharing*”, which focused on the high interest of *prosumers* in energy sharing, like, for instance, interest in selling the extra amount to the general electricity grid (Q44.3) and for the collective consumption of the local community (Q45.2) or an organised form of shared consumption with the local community (Q44.2). It explained 13.095% of the total variance.

Factor 3 was labeled “ *Concern on Climate Change*”, which differentiated from Factor 1 for being saturated by items specifically on climate changing (Q15.1), rising of temperature (Q16.3), and extreme weather conditions (Q16.4), that also presented a mild correlation with Factor 1 (as can be seen in greyed-out values in Table 4). It explained 8.629% of the total variance.

Factor 4 was labeled “ *Social Influence*” for the high tendency of asking for advice before switching to a different energy supply service (Q41) and the availability to adopt renewable energies when the majority of your neighbors are doing so (Q42). It explained 6.772% of the total variance.

Factor 5, finally, was labeled as “ *Impact of Costs on Bills*” as *prosumers* resulted interested in Less (Q34.1) or unvaried costs of bills when adopting a *prosumer* model (Q34.2). This factor explained 5.529% of the total variance of being a *prosumer*.

### Linear regression

Linear regression has been conducted to predict the influence of the independent variables on the dependent variable “Acceptance of being a Prosumer”. A significant regression equation was found F(3,125)=10.668, p>0.01 ( Table 7) with an R ^2^ of .204 ( Table 6) considering a Model with 3 main factors: Overall Attitude Scoring; Economic benefits (e.g. lower energy costs, potential income); and Age ( Table 5).

**Table 5.** Variables Entered ^a^.

Model	Variables Entered	Variables Removed	Method
1	Attitudes overall score	.	Forward (Criterion: Probability-of-F-to-enter <= ,050)
2	Economic benefits (i.e., lower energy costs, potential income).	.	Forward (Criterion: Probability-of-F-to-enter <= ,050)
3	Age	.	Forward (Criterion: Probability-of-F-to-enter <= ,050)

^a.^ Dependent Variable: ACCEPTANCE_Being_a_Prosumer

**Table 6.** Model Summary.

Model	R	R Square	Adjusted R Square	Std. Error of the Estimate	Durbin-Watson
1	.354 ^a^	.125	.119	1.3349	
2	.404 ^b^	.163	.150	1.3111	
3	.451 ^c^	.204	.185	1.2838	1.879

a. Predictors: (Constant), Attitudes. b. Predictors: (Constant), Attitudes, q39_2 Economic benefits (e.g. lower energy costs, potential income). c. Predictors: (Constant), Attitudes, q39_2 Economic benefits (e.g. lower energy costs, potential income), Age d. Dependent Variable: ACCEPTANCE_Being_a_Prosumer_SCORING

**Table 7.** ANOVA ^a^.

Model		Sum of Squares	df	Mean Square	F	Sig.
1	Regression	32,449	1	32,449	18,209	,000 ^b^
	Residual	226,311	127	1,782		
	Total	258,760	128			
2	Regression	42,173	2	21,086	12,267	,000 ^c^
	Residual	216,587	126	1,719		
	Total	258,760	128			
3	Regression	52,746	3	17,582	10,668	,000 ^d^
	Residual	206,013	125	1,648		
	Total	258,760	128			

a. Dependent Variable: ACCEPTANCE_Being_a_Prosumer_SCORING b. Predictors: (Constant), Attitudes c. Predictors: (Constant), Attitudes, q39_2 Economic benefits (e.g. lower energy costs, potential income). d. Predictors: (Constant), Attitudes, q39_2 Economic benefits (e.g. lower energy costs, potential income). , Age

**Figure 5.**
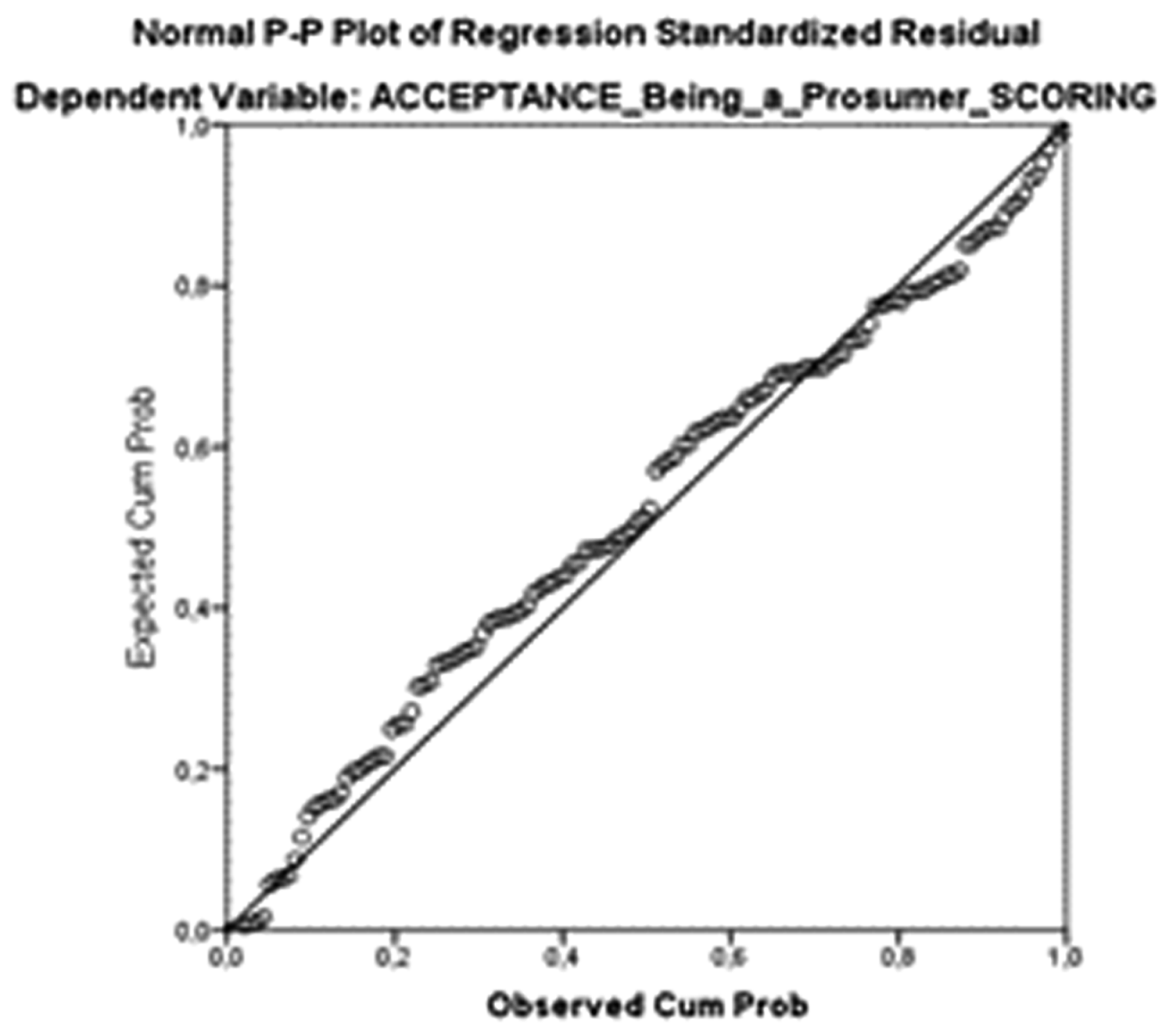
Observed cumulative distribution function of the standardized residual compared to the expected cumulative distribution of the normal distribution.

**Figure 6.**
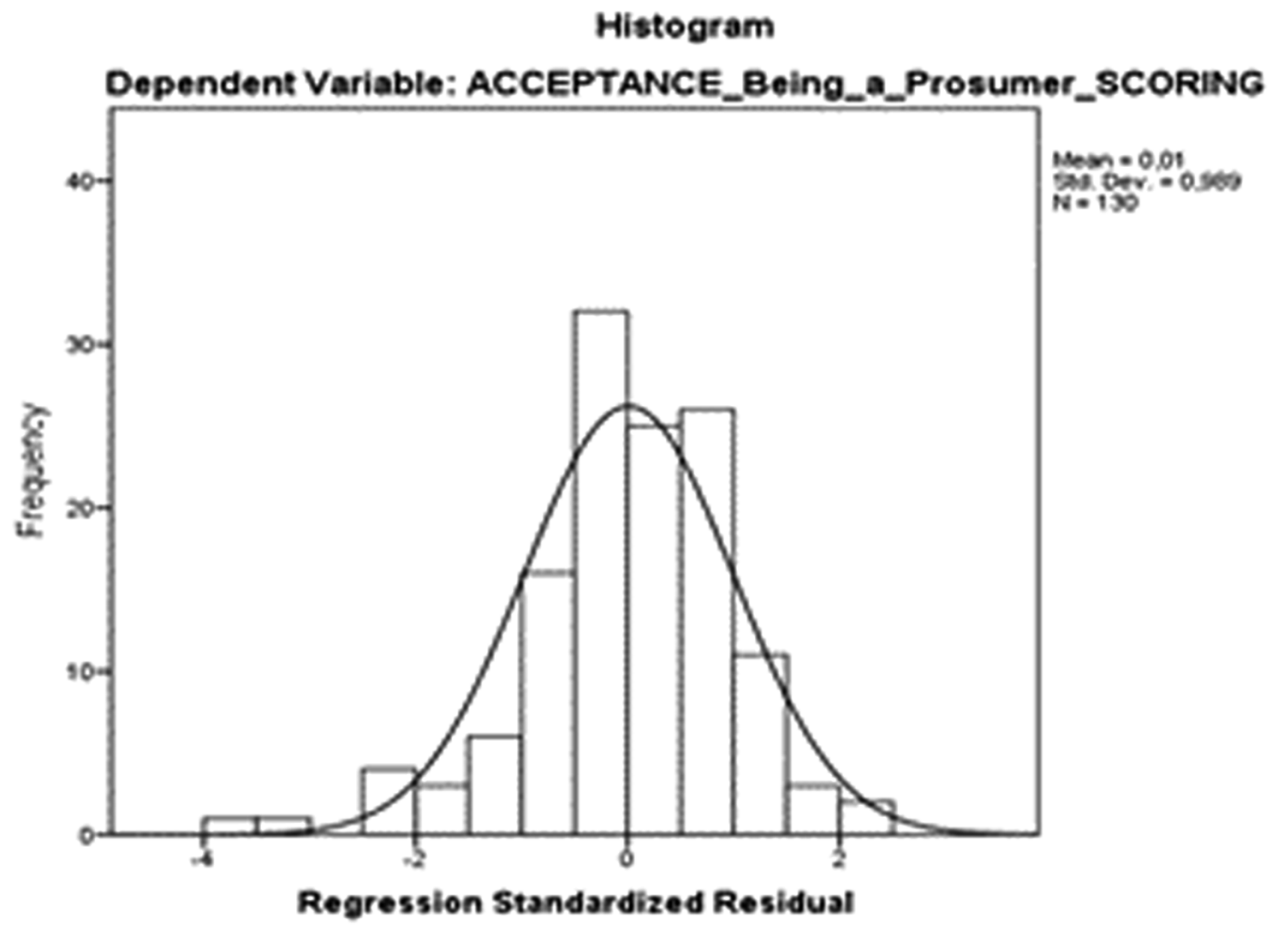
Histogram distribution of standardized residuals values based on the acceptance of being a prosumer model.

Participants predicted acceptance of becoming a *prosumer* was equal to 5.399 (Constant) + .057 (Overall Attitude scoring) + 181 (Perception of Economic Benefit score) - .234 (Age of participant). That is to say that participant acceptance of becoming a *prosumer* increased when attitudes overall scoring, perception of economic benefits, but do decrease with age. Despite R ^2^ being not so powerful (below .300), Collinearity Statistics were acceptable ( Table 8), and both the scatterplot ( Figure 5) and the standardized residuals were normally distributed ( Figure 6).

**Table 8.** Coefficients ^a^.

Model		Unstandardized Coefficients		Standardized Coefficients	t	Sig.	Collinearity Statistics	
		B	Std. Error	Beta			Tolerance	VIF
1	(Constant)	5.069	.882		5.748	.000		
	Attitudes	.059	.014	.354	4.267	.000	1.000	1.000
2	(Constant)	4.728	.878		5.386	.000		
	Attitudes	.058	.014	.346	4.238	.000	.998	1.002
	Economic benefits (e.g. lower energy costs. potential income).	.165	.069	.194	2.378	.019	.998	1.002
3	(Constant)	5.399	.900		6.002	.000		
	Attitudes	.057	.013	.342	4.275	.000	.998	1.002
	Economic benefits (e.g. lower energy costs. potential income).	.181	.068	.212	2.649	.009	.990	1.010
	Age	-.234	.092	-.203	-2.533	.013	.992	1.009

a. Dependent Variable: ACCEPTANCE_Being_a_Prosumer_SCORING

## Discussion

The present research aimed at identifying psychological factors that could predict social acceptance becoming a *Prosumer*. Specifically, knowledge, awareness levels, attitude, control perception, and norms could influence the intention to adopt new RETs business models. Starting from the three Wüstenhagen *et al.* (2007) dimensions, a fourth dimension with a more direct focus on the individual characteristics has been added, including TPB, TAM, NAM, EA and Environmental risk perception. A structured survey using consolidated items measuring different psychological variables involved in becoming a *prosumer* was generated and thus the latent factors were highlighted, thanks to exploratory factorial analysis. To predict the acceptance scoring, linear regression has been performed, to identify which specific questions could be used in future research to predict social acceptance of becoming a *prosumer*. Results clearly state the latent factors behind the availability of being a *prosumer* and provide an indication to which items could be selectively being included in surveys to measure the intended behavior of becoming a *prosumer*.

### Significant factors behind potential prosumers

Through Factorial Analysis it was possible to: identify the main individual and social factors able to intervene on the intention of becoming a *prosumer* and; quantify their importance. The principal axis Factorial Analysis on the N=112 “ *prosumers*” of the sample, revealed 5 latent psychological factors:

- **Factor 1. Concern about environmental issues**: the potential prosumer has an accurate and articulate knowledge about different types of environmental issues such as knowledge of acidification of rains and oceans, loss of biodiversity, air pollution, traffic congestion or waste disposal, etc. The prosumer is aware of global environmental issues and with strong beliefs and pro-environmental attitudes about it, which contribute to rise her/his concerns about environmental issues.
- **Factor 2. Interest in energy sharing**: the potential prosumer is favorable in accepting a RET when she/he is interested in energy sharing solutions for the collective consumption of the local community, as a way to reduce the individual environmental impact and improve the local community in a sustainable way, especially in terms of economic incentives where the extra amount can be sold to the general electricity grid.
- **Factor 3. Concern on climate change**: the potential prosumer is not only concerned about environmental issues, but she/he is well aware of the consequences of such issue on climate change, especially on a global scale level, such as the rising of the temperatures and extreme weather conditions. The prosumer develops pro-environmental attitudes specifically about the global climate change course and is sensitive about possible behavior aimed at mitigate such human impact;
- **Factor 4. Social Influence**: The prosumer, as a group member of a local community, is potentially prone to look for support from peers. It is no surprise then when it comes to RET, she/he could be prone to look for advice from significant other and to internalize social norms that are perceived common of her/his national and geographical region (e.g., based on shared laws or cultural stereotypes).
- **Factor 5. Impact on bill cost**: from a more pragmatic point of view, the potential prosumer is prone to adopt a new RET when economic incentives are perceived. This economic aspect seems to affect the decision-making process specifically in a situation that could potentially result in less expensive bills or unvaried bills.

These 5 factors represent data-driven aspects that can be coherently being reconducted to the psychological theories chosen as reference. Latent to social acceptance is the awareness ( Figure 3 – element II) of environmental issues and climate change; environmental attitude ( Figure 3 – element III), formed by concerns and beliefs, in the direction of acting PEBs through behavioral intention ( Figure 3 – element VI); the development of social norms ( Figure 3 – element II), based on community/nation‘s social influences, to engage only certain types of behaviors; and the perceived behavioral control ( Figure 3 – element IV) as balancing between perceived pros and cons, benefits and risks, in the decision-making process, where the economic incentive is the brief-term benefit usually not observable with these types of energies.

### Predict the acceptance of renewable energy technology models

Once described five-factors psychological model of “prosumer” social acceptance, the model was tested in order to quantify the ability of the factors in predicting the acceptance of RET on the entire sample. An overall scale measuring “acceptance of a new energy solution” was used as dependent variable, and it was possible to quantify the contribution of the five factors measuring: awareness of environmental issues and climate change; environmental attitudes; social influence; and environmental risk perception. From the linear regression, it was possible to assert that three variables are able to significantly measure and predict the scores of the “Acceptance in becoming a *prosumer*” ad hoc scale.

### Variable 1: Attitudes

To predict someone's acceptance in becoming a *Prosumer*, fundamental is to consider their attitudes. In the Prosumer Index, attitudes were measured, as intended in TPB ( Ajzen, 1991), as the degree to which a person has a favorable or unfavorable evaluation or appraisal of the behavior. Within the model, it was found as a predictor of the behavioral intention to accept and adopt a new RET business model such as the *prosumer* one. Specifically, EA ( Hines *et al.*, 1987), as the feelings on particular aspects of the environment or objects related to the environment, predicts the behavioral intention towards acceptance. Furthermore, to have favorable or unfavorable attitudes, the subject bases the evaluation on knowledge and awareness. Attitudes were operationalized and measured as the agreement to specific environmental issues and global climate change issues to assess their concern and evaluation in the direction of a behavioral intention. People characterized by high scores in environmental attitudes and detailed knowledge on environmental issues (such as biospheric values), are the ones with most favorable scores about moving towards a different energy solution. They should be the first members of communities to be involved in such process, in order to facilitate and trigger also the positive social influence of other potential prosumer living in their local community.

### Variable 2: Economic incentive

The sensitivity toward the economic incentive is the second element able to predict someone’s behavioral intention to adopt a new RET. In the research, this aspect was reconducted to the perceived behavioral control ( Ajzen, 1991) and its related Environmental Risk Perception, in terms of perceived short-term benefits (usually absent in RET solutions as identified by Wüstenhagen *et al.*, 2007) and long-term costs, both part of the decision-making process in terms of expected outcomes ( Bandura, 1977) of the behavior itself. Strategies or policies that meet such economic characteristic, such as lower or invariant bill’s energy costs, are linked to higher scores in the acceptance of such business models. Such link, appear significant regardless of the presence of other economic indicators, investigated in the present survey (e.g. average income, place of residency, family members etc.). According to Ryan & Deci (2000) in the Self-Determination theory “extrinsic motivation” triggered by economic incentives, would work as classic “reinforcement” that can motivate the people with less score in pro-environmental attitudes, in becoming a prosumer. At the same time, “intrinsic motivation” would find satisfaction by fulfilling values and psychological needs (such as healthy and sustainable behaviors) that enable also positive expected outcomes such as controlling energy costs and contribution the local community. Indicating that the psychological mechanism behind the economic aspect of “prosumer” acceptance should dwell into the perceived sense of control (e.g., positive expected outcomes of the pro-environmental behavior) and the consequent (intrinsic or extrinsic) motivation process.

### Variable 3: Age

Age is the third variable that was found to predict the acceptance in becoming a *prosumer*. Consistently with previous research, younger people seemed to be more sensitive to environmental dimensions and issues as opposed to older adults. Younger respondents in our sample had fewer economic possibilities compared to adults. Their smaller economic possibilities don’t seem to influence their already established view and perception of the energy supply. Although younger people can be seen still in the process phases of auto-determination in terms of attitudes and norm, and they are more subject to peer and community influences, it is fair to assume that their LOC orientation ( Rotter, 1966) tends to be more internal, perceiving their course of action as a consequence of their personal decisions. Hines *et al.* (1987), for instance, showed that LOC is one of the personality factors to determine PEBs intention.

To conclude, any stakeholder, policy maker or regulator that envision the creation of surveys and questionnaires favoring/facilitating or supporting citizens' acceptance transition of a RET, and for example, in becoming a *prosumer*, these three factors should be measured to consider, treading the path towards those individuals prone to accept and adopt a RET business model.

### Strengths, limitations, and future research

Various theories used in this research have already been profoundly studied and presented in different papers and journals, however the *prosumer* business models, as RETs, is a relatively new concept where scant literature is available, as it also is in the European reality, where such a typology of energy supply is almost completely nil. This point gives strength to the present research in the attempt to put the basis for the understanding of individual acceptance related to broader social acceptance. Furthermore, trying to understand the individual acceptance, the research tries to define who is the *prosumer* from a specific psychological point of view, and how this psychological dimension influences RETs adoption, highlighting the existing relation between the subject and the new sustainable business models. This opens the road to better understanding and defining which are the overall characteristics and conditions to foster such a transition towards sustainability.

Regarding limitations, the research stops on a “behavioral intention” (VI) level and does not take into exam observable actual behaviors.

Second, the answer given to the survey may be biased by the framing of the questions, for example, not mentioning in detail what “collective and shared” consumptions stand for and unavoidably failing in describing a large amount of different possible solutions.

Hidden costs, transparency issues, and low maturity of services are further aspects to be investigated in order to have a more comprehensive understanding of Social Acceptance as possible risks for respondents.

Regarding the sample, it is neither representative of the European (EU-27) population, nor a specific Nation or specific social group. The respondents have been collected among members involved in European discussion tables of new energy business models and renewable energy technologies.

Due to the project requirements and scopes and the model used in the paper, different psychological aspects of the person have been just mentioned or left out. For instance, personality traits are fundamental characteristics to investigate in an environmentally concerned person; important is the trustworthiness in the other actors such as policymakers, marketers, etc., and in the technology itself. Two dimensions of trust have been identified by research and are particularly relevant for project acceptability, namely competence-based trust (i.e., trust in knowledge and expertise of responsible agents) ( Gordon *et al.*, 2014; Terwel *et al.*, 2009) and integrity-based trust (i.e., trust in honesty and transparency of responsible agents) ( Braun *et al.*, 2018; Graham *et al.*, 2009). Fundamental is to consider the environment the subject lives in and what are the policies regarding renewable energies in the country; and finally, related to these last two aspects is the need of a better understanding of the community point of view accepting a RET, not as the singular individual.

Future research will be able to focus on actual behaviors of accepting and adopting such RETs. Other intervening psychological individual factors such as trust, personality, community-specific factors and dynamics could be explored to integrate the already existing factors.

Future research will be able to study the above-mentioned characteristics gathering representative samples of national and European communities, using this research as a baseline to be adapted for specific community/nation groups, and exploring further individual psychological elements.

## Conclusions

The present research gave an overview of the already identified dimensions of social acceptance with the introduction of a fourth dimension specific to individuality and its psychological dimensions, focusing on social and individual factors that influence behavioral intention to accept a RET. This research can facilitate policymakers and relevant stakeholders to better understand which relevant psycho-sociological factors are intervening in these processes and what and how specifically target when proposing change towards sustainable energy production and consumption.

For the ones that are already *prosumers*, five latent factors have been identified: what drives the change in accepting and adopting a RET is the concern about environmental issues and the concern on climate change. The individual is indeed interested in sharing the energy production and consumption, and has an interest in an unvaried or lower cost of bills.

Regarding the predictors for the acceptance from individuals to become a *Prosumer*, the linear regression model suggests that at the increasing of Environmental Attitudes, Economic incentives, and decreasing of people’s Age, individuals are prone to accept and adopt a new RET business model like the *prosumer* business models. Measuring Attitude proper to the individual, what are his knowledge, concerns, values, and levels of awareness regarding renewable energies can predict the overall acceptance of becoming a prosumer.

The economic incentives help frame the perception of a possible transition, reducing the usually perceived short-term costs for the individual in juxtaposition to the perceived long-term benefits for the individual, for the local community and for the environment. The economic incentive can provide a positive element for motivation fulfilling the extrinsic (for the reinforcement) and intrinsic motivation in acting a coherent behavior (experiencing competence, autonomy, behaving pro-environmentally).

### Citizen engagement to become prosumer

When coming to propose a new energy business model, it is important to involve the community, its members, and their identity, observing the presence of specific environmental attitudes shared among the group. From the descriptive statistics, friends and colleagues are considered by far the most reliable source for advice, followed by publications and academic journals. Raising the levels of awareness should be a priority through informational campaigns highlighting the agency capacity of these communities if they had to adopt a shared energy plan. The decision-making process should be accompanied by a clear and transparent explanation and framing of the consequences of opting out or opting in with clear benefits and costs. For a positive acceptance, the perceived short-term costs of a RET; the possibility of a landscape disfigurement (depending on the solution); the long-term benefits, etc., should all be explained. Anyhow, knowledge alone is not sufficient. The process should be composed with some behavioral guidelines framed enhancing the advantage of these actions, opposed to negative consequences. Economic incentives, however, should be contemplated by policies and governments, as a boost to fasten the transition, thanks to the reduction of some limiting short-term costs giving the freedom to focus on the long-term benefits and raising satisfaction levels that can fill subjects’ motivation in the decision-making process. Indeed, economic incentives are a strong driver, nevertheless, the participation in the energy transition is driven more by the intention to energy sharing and collective benefits rather than a mere investment.

Other individuals not interested in these initiatives should not be forgotten. The so-called late adopters will be pulled aboard seeing that nations, governments, policies, economic incentives, and citizens are moving in the same direction, undergoing social pressure and finding a mismatch between their personal norms and the line taken and desired, in accordance with the U-curve of Acceptance.

Concerning results and recommendations addressed to policymakers, regulators, and all energy market actors, the present paper suggests expanding research about decision-making processes and the related expectations of involvement by communities and citizens:

- Improving the general level of knowledge and awareness concerning mutual dependences of environmental issues, economical aspects, and energy value chain;
- Identifying the gaps between the actual regulatory landscape and comprehensible, clear, and updated information;
- Increasing the accessibility and effectiveness of information regarding energy transition incentives;
- Supporting the implementation of innovative energy business models and capacity buildings both in the public and private sector by promoting consensus-building across local administrations, energy market actors, and consumers.
- Consider ad-hoc campaigns to involve and raise awareness in adults and incentives and financing for youngers with fewer economic availabilities.

## Data Availability

Zenodo: Predicting Acceptance and Adoption of Renewable Energy Community solutions: The Prosumer Psychology, https://doi.org/10.5281/zenodo.6839681. ( Brambati *et al.*, 2022) This project contains the following underlying data: Database Prosumer psychology - Open research europe.xlsx Zenodo: Predicting Acceptance and Adoption of Renewable Energy Community solutions: The Prosumer Psychology, https://doi.org/10.5281/zenodo.6839681. ( Brambati *et al.*, 2022) This project contains the following extended data: 1st survey RENNAISANCE - LISTA DOMANDE.pdf OUTPUT-SPSS_full_Prosumer psychology.xlsx Data are available under the terms of the Creative Commons Attribution 4.0 International license (CC-BY 4.0).
