# Supplementary material for: Predicting acceptance and adoption of renewable energy community solutions: the prosumer psychology
Source: Open Res Eur. 2022 Sep 29;2:115. [Version 1] doi: 10.12688/openreseurope.14950.1 (PMC10539723; doi:10.12688/openreseurope.14950.1)
Supplement: Peer review history

# Reviewer report: Reviewer response for version 1

**DOI:** 10.21956/openreseurope.16157.r34997

**Published:** 2023-10-17

**Authors:** Jens Lowitzsch, Lucas Roth

**Affiliations:** 1 Faculty of Business Administration and Economics, European University Viadrina, Logenstraße, Germany; 2 Faculty of Business Administration and Economics, European University Viadrina, Frankfurt an der Oder, Brandenburg, Germany

We use the plural "we" as I have reviewed this article together with my assistant, Lucas Roth, who is familiar with the topic.

 The open letter touches upon an interesting topic. It seeks to analyse enabling psychological dimensions that potentially support the energy transition by shifting from what the authors refer to as a "technological-driven" to a "consumer-driven" approach. Subsequently, the authors develop a "prosumer acceptance index" that reflects purely psychological factors influencing the acceptance of renewables. The analysis was conducted using data gathered through an online questionnaire platform. The authors used factor analysis paired with an ordinary least square regression to quantify the influence of identified factors. In a nutshell, the researchers identify five latent factors that drive the acceptance of renewable energy technology: concern about environmental issues, energy-sharing practices, and interest in constant or lower electricity bills. The best predictors for the acceptance model are environmental attitude, economic incentives and age.

 The presented work is certainly of great value to the scientific community. The focus on enabling factors to increase the acceptance of renewables is now more than ever one of the biggest scientific and practical challenges in the energy transition. Therefore, we thank the authors for their contributions and perspectives. We believe that the research article has merit in its current version. However, we suggest improvements listed in the comments below.

 Comments:

1. The abstract holds the most important information, but the clarity could be improved. The first sentence alone holds five times the word "and" which makes it hard to follow. The analysis issues could be separated to facilitate the way the information is presented. Moreover, the causality could be brought out better. Currently, the authors start with: "(…) aims at supporting a data-driven approach able to deal with climate change and identify and quantify the psychosociological dimensions and factors that could support the transition from a technology-driven approach to a consumer-driven approach (…)." In fact, I would argue that the researchers primarily identify enabling factors to increase technological acceptance. This consumer-driven approach potentially facilitates the energy transition, contributing to combating climate change. Furthermore, tension between technology-driven and consumer-driven approaches in the energy transition is a significant challenge. However, the authors do not reflect on this circumstance in the course of their research while specifically mentioning it in the abstract. The implications of the own work should be brought out more in this respect later in the manuscript. Lastly, the phrase "This research can facilitate policymakers (…)" should be edited. The research helps policymakers or provides guidance. This fragment reappears on p. 7 and p. 16.
2. I think the manuscript would benefit from separating the introduction from a part on the theoretical background and a literature review. I am also missing a thorough review of literature that looks specifically at the issue the authors are analysing. In this respect, much has been done using similar approaches. The literature analysis under "Individual acceptance: what does psychology say about prosumers?" (p. 5) uses mostly old references. That is not bad per se, but it could be supplemented by newer work from the field. To name a few: Conradie *et al.* (2021 ^1^), Chen (2016 ^2^), Liobikienė *et al.* (2021 ^3^), Bhutto *et al.* (2020 ^4^).
3. I am unsure about the data collection. How was the sample identified? How many invitations were sent out (response rate)?
4. The methodological part should mathematically reflect the index construction. Moreover, the factor analysis and the regression model should be mathematically reflected. This part is missing. The authors may also do this in the appendix. But it would help to understand what has been done better from a methodological perspective.
5. The appendix should include a complete version of the questionnaire with an English translation.
6. The methodological limitations should be reflected in its own section. Here, the researchers should pinpoint potential statistical restrictions of their research better. The sample is with 212 respondents small. We further see that due to missing data, information on several dimensions is very limited (e.g., p. 9, information on social norms comes only from 131 participants). A better reflection on the sample selection would improve the understanding of potential sampling errors better. For factor analysis, the small sample has several implications (e.g., low power, unstable or inaccurate loadings, overfitting). This should be reflected. Similarly, a regression with a small sample size will likely have large confidence intervals. Larger samples and narrower CIs might lead to a different picture. The presented results certainly have merit, but possible flaws should be pointed out better. Diagnostics depicted in the appendix could build further trust in the analysis.
7. The conclusions under "Citizen engagement to become prosumer" (p. 17) are very general and not well tied to the findings of the conducted research. This should be improved.
8. The relationship to the RENISSANCE project is not clear to us. Please elaborate on this. On p. 3, you write, "Different business models for sustainable community energy production and consumption can be identified, and amongst them, RENAISSANCE identified a set that we can call "Prosumer business models" (RENAISSANCE, 2020)." How does that tie in with your research?
9. Clearer images of the residual plot and the histogram in Figures 4 and 5 would benefit the overall quality. (p. 14).

Again, we thank the authors for their interesting work. All in all, the authors present compelling information important to the field. We believe that after the above revisions, the paper is suitable for indexing.

If applicable, is the statistical analysis and its interpretation appropriate?

Yes

Is the study design appropriate and is the work technically sound?

Yes

Is the work clearly and accurately presented and does it engage with the current literature?

Partly

Are the conclusions drawn adequately supported by the results?

Partly

Are sufficient details of methods and analysis provided to allow replication by others?

Partly

Are all the source data and materials underlying the results available?

Yes

Reviewer Expertise:

Prof. Jens Lowitzsch is a policy expert in the renewable energy transition and experienced in quantitative study designs. Lucas Roth works as an applied econometrician.

We confirm that we have read this submission and believe that we have an appropriate level of expertise to confirm that it is of an acceptable scientific standard, however we have significant reservations, as outlined above.

## References

[1] : Who wants to join a renewable energy community in Flanders? Applying an extended model of Theory of Planned Behaviour to understand intent to participate. *Energy Policy* .2021;151: 10.1016/j.enpol.2020.112121 10.1016/j.enpol.2020.112121

[2] : Extending the theory of planned behavior model to explain people's energy savings and carbon reduction behavioral intentions to mitigate climate change in Taiwan–moral obligation matters. *Journal of Cleaner Production* .2016;112: 10.1016/j.jclepro.2015.07.043 1746-1753 10.1016/j.jclepro.2015.07.043

[3] : The determinants of renewable energy usage intentions using theory of planned behaviour approach. *Renewable Energy* .2021;170: 10.1016/j.renene.2021.01.152 587-594 10.1016/j.renene.2021.01.152

[4] : Adoption of Energy-Efficient Home Appliances: Extending the Theory of Planned Behavior. *Sustainability* .2020;13(1) : 10.3390/su13010250 10.3390/su13010250

# Reviewer report: Reviewer response for version 1

**DOI:** 10.21956/openreseurope.16157.r35002

**Published:** 2023-09-28

**Authors:** Yasir Ahmed Solangi

**Affiliations:** 1 Nanjing University of Aeronautics and Astronautics, Nanjing, Jiangsu, China; 2 School of Management, Jiangsu University, Zhenjiang, Jiangsu, China

The article presents a comprehensive study on the social acceptance of renewable energy and the transition to prosumer (producer-consumer) business models. It aims to identify and quantify psycho-sociological dimensions and factors influencing the adoption of these models, ultimately creating a Prosumer Acceptance Index. The study employed questionnaire data collected through an online survey platform, conducted factorial analysis to reveal latent psychological factors, and performed linear regression to predict behavioral intentions. The results identified five psychological factors and three variables that significantly measure prosumer acceptance. The research has practical implications for policymakers and stakeholders in promoting sustainable energy production and consumption. The article is well-structured and addresses its research objectives effectively.

If applicable, is the statistical analysis and its interpretation appropriate?

Yes

Is the study design appropriate and is the work technically sound?

Yes

Is the work clearly and accurately presented and does it engage with the current literature?

Yes

Are the conclusions drawn adequately supported by the results?

Yes

Are sufficient details of methods and analysis provided to allow replication by others?

Yes

Are all the source data and materials underlying the results available?

Yes

Reviewer Expertise:

The article addresses an important and timely topic within my research area, which focuses on the social acceptance of renewable energy and the transition to prosumer business models.

I confirm that I have read this submission and believe that I have an appropriate level of expertise to confirm that it is of an acceptable scientific standard.

# Reviewer report: Reviewer response for version 1

**DOI:** 10.21956/openreseurope.16157.r31630

**Published:** 2023-06-16

**Authors:** Ruth Carbajo

**Affiliations:** 1 Universidad de Lleida, Lleida, Spain

This paper broadly describes the dimensions engaged in the process of acceptance in terms of consumers driven approaches and prosumers beyond the already existing considerations. It presents a Prosumer Acceptance Index to identify and quantify individual psychological dimensions to understand the processes and factors that underlie individual acceptance and adoption of new renewable energy business models. For the construction of the index, two steps are considered:

 1) Identify the main factors (individual and social) behind the adoption of being a *prosumer* and quantify their importance (for the ones that were already *prosumers*).

 2) Identify and quantify the factors that could be better able to predict the decision to become a *prosumer* (for the ones that are not yet *prosumers.*

 The work is presented in an accurate manner, starting from a contextualization within the body of literature from the variety of renewable energy-based business models to the consideration of the social acceptance dimensions within RETs. In this review, the theoretical definitions of the *prosumer* psychological individual and social characteristics leading to social acceptance of this new renewable energy business model are collected.

 The study design is appropriate and the work technically sound, with sufficient details of methods and analysis provided to allow replication. All the source data and materials underlying the results are available and the statistical analysis and its interpretation is appropriate. I will highlight the construction of models and the consideration of the factors.

 The conclusions are adequately supported by the results, but as happened on this type of projects, sometimes the argumentative weight of the conclusions relies more on elements drawn from the literature review than from the conclusions of the analysis of the survey process. Moreover, the total of respondents is low.

If applicable, is the statistical analysis and its interpretation appropriate?

I cannot comment. A qualified statistician is required.

Is the study design appropriate and is the work technically sound?

Yes

Is the work clearly and accurately presented and does it engage with the current literature?

Yes

Are the conclusions drawn adequately supported by the results?

Yes

Are sufficient details of methods and analysis provided to allow replication by others?

Yes

Are all the source data and materials underlying the results available?

Yes

Reviewer Expertise:

Ethics of technology, energy trasition, RRI and enrgy communities

I confirm that I have read this submission and believe that I have an appropriate level of expertise to confirm that it is of an acceptable scientific standard.

## References

[1] : A typology for unpacking the diversity of social innovation in energy transitions. *Energy Research & Social Science* .2022;88: 10.1016/j.erss.2022.102513 10.1016/j.erss.2022.102513
